# Genomic characterization of Sabiá virus in Brazil, 2019–2020: Implications for diagnostics, virus evolution, and receptor binding

**DOI:** 10.1371/journal.pntd.0014008

**Published:** 2026-02-20

**Authors:** Ingra M. Claro, Erika R. Manuli, Camila A. M. da Silva, Thaís M. Coletti, Philippe Lemey, Ana Catharina Nastri, Luciana Vilas Boas Casadio, Amaro Nunes Duarte-Neto, Joshua Quick, Camila M. Romano, Charles Whittaker, Sarah C. Hill, Carlos A. Prete, Darlan S. Candido, Filipe R. R. Moreira, Mariana S. Ramundo, Ian Nunes Valença, Jaqueline G. de Jesus, Flavia C. S. Sales, Mariana S. Cunha, Juliana M. Guerra, Maria Cassia Mendes-Correa, Tania R. Tozetto-Mendoza, Marcilio Jorge Fumagalli, Yeh-Li Ho, Peter Simmonds, Weng M. Ng, Thomas A. Bowden, William M. de Souza, Oliver G. Pybus, Anna S. Levin, Nicholas Loman, Ester C. Sabino, Nuno R. Faria

**Affiliations:** 1 Departamento de Moléstias Infecciosas e Parasitárias, Faculdade de Medicina da Universidade de São Paulo, São Paulo, Brazil; 2 Instituto de Medicina Tropical, Faculdade de Medicina da Universidade de São Paulo, São Paulo, Brazil; 3 School of Public Health, Imperial College London, MRC Centre for Global Infectious Disease Analysis and the Abdul Latif Jameel Institute for Disease and Emergency Analytics (J-IDEA), London, United Kingdom; 4 Department of Microbiology, Immunology and Molecular Genetics, College of Medicine, University of Kentucky, Lexington, Kentucky, United States of America; 5 Universidade Municipal de São Caetano do Sul, São Caetano do Sul, São Paulo, Brazil; 6 Department of Microbiology, Immunology and Transplantation, Rega Institute, KU Leuven – University of Leuven, Leuven, Belgium; 7 Divisão de Doenças Infecciosas, Hospital das Clínicas, Faculdade de Medicina, Universidade de São Paulo, São Paulo, Brazil; 8 Departamento de Patologia, Faculdade de Medicina da Universidade de São Paulo, São Paulo, Brazil; 9 Núcleo de Anatomia Patológica, Instituto Adolfo Lutz, São Paulo, Brazil; 10 School of Biosciences, University of Birmingham, Birmingham, United Kingdom; 11 Department of Biology, University of Oxford, Oxford, United Kingdom; 12 Department of Pathobiology and Population Sciences, Royal Veterinary College, Hatfield, United Kingdom; 13 Departamento de Engenharia de Sistemas Eletrônicos, Escola Politécnica da Universidade de São Paulo, São Paulo, Brazil; 14 Departamento de Genética, Instituto de Biologia, Universidade Federal do Rio de Janeiro, Rio de Janeiro, Brazil; 15 Laboratório de Imunologia, INCOR, Hospital das Clinicas HCFMUSP, Faculdade de Medicina, Universidade de São Paulo, São Paulo, Brazil; 16 Departamento de Clínica Médica, Disciplina de Imunologia Clínica e Alergia, Faculdade de Medicina da Universidade de São Paulo, São Paulo, Brazil; 17 Centro de Virologia, Núcleo de Doenças de Transmissão Vetorial, Instituto Adolfo Lutz, São Paulo, Brazil; 18 Centro de Pesquisa em Virologia, Faculdade de Medicina de Ribeirão Preto, Universidade de São Paulo, Ribeirão Preto, Brazil; 19 Research Complex at Harwell, Harwell Science and Innovation Campus, Didcot, United Kingdom; 20 Electron Bio-Imaging Centre, Diamond Light Source, Didcot, United Kingdom; 21 Division of Structural Biology, Centre for Human Genetics, University of Oxford, Oxford, United Kingdom; 22 Department of Infection Control, Hospital das Clínicas, Faculdade de Medicina, Universidade de São Paulo, São Paulo, Brazil; Center for Disease Control and Prevention, UNITED STATES OF AMERICA

## Abstract

Between December 2019 and January 2020, two patients suspected of having severe yellow fever were admitted to a tertiary healthcare facility in São Paulo, Brazil, presenting with acute hemorrhagic syndrome and neurological alterations; both cases had fatal outcomes. Upon admission, both tested negative for yellow fever viral RNA, and Sabiá virus (SABV), a New World arenavirus, was identified as the causative pathogen. To date, only four humans naturally acquired SABV infections have been confirmed, all fatal and linked to rural settings. We applied next-generation sequencing to generate complete and near-complete genomes from two patients (SP17 and SP19). Existing molecular diagnostics failed to detect SABV; therefore, new molecular tests were developed. Genetic analyses of SP17 and SP19 genomes along with other arenaviruses, revealed that the new cases were genetically diverse, showing 93-98.2% amino acid identity at the NP level among SP17, SP19, and the 1990 reference strain (SPH114202). Time-scaled phylogenetic analyses confirmed that SP17 and SP19 were not epidemiologically linked and suggested that SABV has been circulating undetected in Brazil for over a century. Additionally, homology modeling and structure-based mapping provided insights into SABV receptor-binding sequence conservation, suggesting that SABV shares similar receptor binding structure to other clade B arenaviruses, despite some amino acid variation around receptor binding site. Our findings underscore the need for retrospective and prospective surveillance of undiagnosed hemorrhagic fever cases to assess the public health impact of SABV in Brazil.

## Author summary

Sabiá virus (SABV), also known as *Brazilian mammarenavirus*, causes severe hemorrhagic fever, with mortality rates among New World arenaviruses averaging around 30%. Over the past 40 years, four naturally acquired cases have been confirmed in Brazil, all fatal and linked to rural settings. Here, we characterized the genomic features of the third and fourth naturally acquired cases, identified in 2019 and 2020 in São Paulo, Brazil, in patients initially suspected of yellow fever. These cases were detected using untargeted metagenomic sequencing in a São Paulo healthcare facility. Our findings reveal that existing molecular diagnostics failed to detect contemporary SABV strains, leading to the development of novel assays based on newly generated SABV genomes. Genetic analyses confirm that these cases were not epidemiologically linked and provide evidence of SABV circulation in Brazil for over a century. Additionally, we present new insights into SABV receptor interactions, improving our understanding of its tropism. These findings expand the limited knowledge of SABV epidemiology and genetic diversity, with direct implications for diagnostics, surveillance, and outbreak preparedness against this highly pathogenic virus. Greater efforts should focus on pathogen-agnostic investigations of hemorrhagic fever cases, particularly in patients who test negative for yellow fever. Expanded surveillance is essential to better understand the disease spectrum, pathogenicity, transmissibility, and zoonotic reservoir of SABV.

## Introduction

Sabiá virus (SABV) is an enveloped negative-stranded, bi-segmented RNA virus that is classified as a *Brazilian mammarenavirus*, *Arenaviridae* family [1]. Within the family, SABV belongs to Clade B from the New World arenaviruses complex (NWA) [2]. This clade contains other highly pathogenic threats known to cause severe hemorrhagic fever disease, including Chaparé (CHHF), Machupo (MACV), Junín (JUNV), and Guanarito (GTOV) viruses [2]. Like other mammarenaviruses, SABV contains two single-stranded, ambisense RNA segments: the small (S) segment (~3,500 nt) encodes the envelope glycoprotein precursor (GPC) and the nucleoprotein (NP), while the large (L) segment (~7,200 nt) encodes the zinc-binding matrix protein (Z) and the viral RNA-dependent RNA polymerase (RdRp) [3].

SABV was first identified in 1990 in the municipality of Cotia, São Paulo state, Brazil [2]. Nearly a decade later, in 1999, a second human infection was identified in Espírito Santo do Pinhal, about 155 km north of Cotia, also in São Paulo state [4]. Additionally, two laboratory-associated infections occurred following accidental aerosol exposure, one in 1992 (Brazil) and another in 1994 (USA). The 1992 case recovered with supportive care alone, without antiviral treatment, whereas the 1994 case was treated with ribavirin and recovered; however, a causal role of ribavirin in clinical recovery cannot be established based on this single case [5,6].

Our understanding of SABV evolution, pathogenicity, and reservoir remains limited due to insufficient data, partly resulting from the lack of specific diagnostic tools [7] and associated biohazard risks. Work with this virus requires biosafety level 4 (BSL-4) facilities, which are currently unavailable in South America. Although the tropism of viruses within the NWA complex is varied, MACV, JUNV, GTOV and SABV seem to utilize the human transferrin receptor (hTfR1) for entry into host cells [8]. Arenaviruses are primarily transmitted through aerosols, with wild rodents acting as the principal reservoirs. Human infection typically occurs via inhalation of aerosols contaminated with rodent excreta [9].

In this study, we investigated two SABV infections identified in patients hospitalized with severe hemorrhagic fever initially suspected of yellow fever at São Paulo’s main tertiary hospital between December 2019 and January 2020. Using viral genomic data generated directly from clinical samples, we examine the evolutionary history of contemporary SABV strains and assess whether genetic divergence from the historical reference strain has implications for molecular detection and receptor usage.

## Results

Untargeted metagenomic sequencing using the SMART-9N metagenomic approach, confirmed the presence of SABV RNA in patient SP17 [10,11], a 52-year-old male from Sorocaba, SP, Brazil, with a history of forest hiking who sought care at a basic health facility in SP on December 30, 2019. His condition worsened, leading to referral to the *Hospital das Clínicas* on January 6, 2020, where the patient died on January 11, 2020.

**Fig 1:**
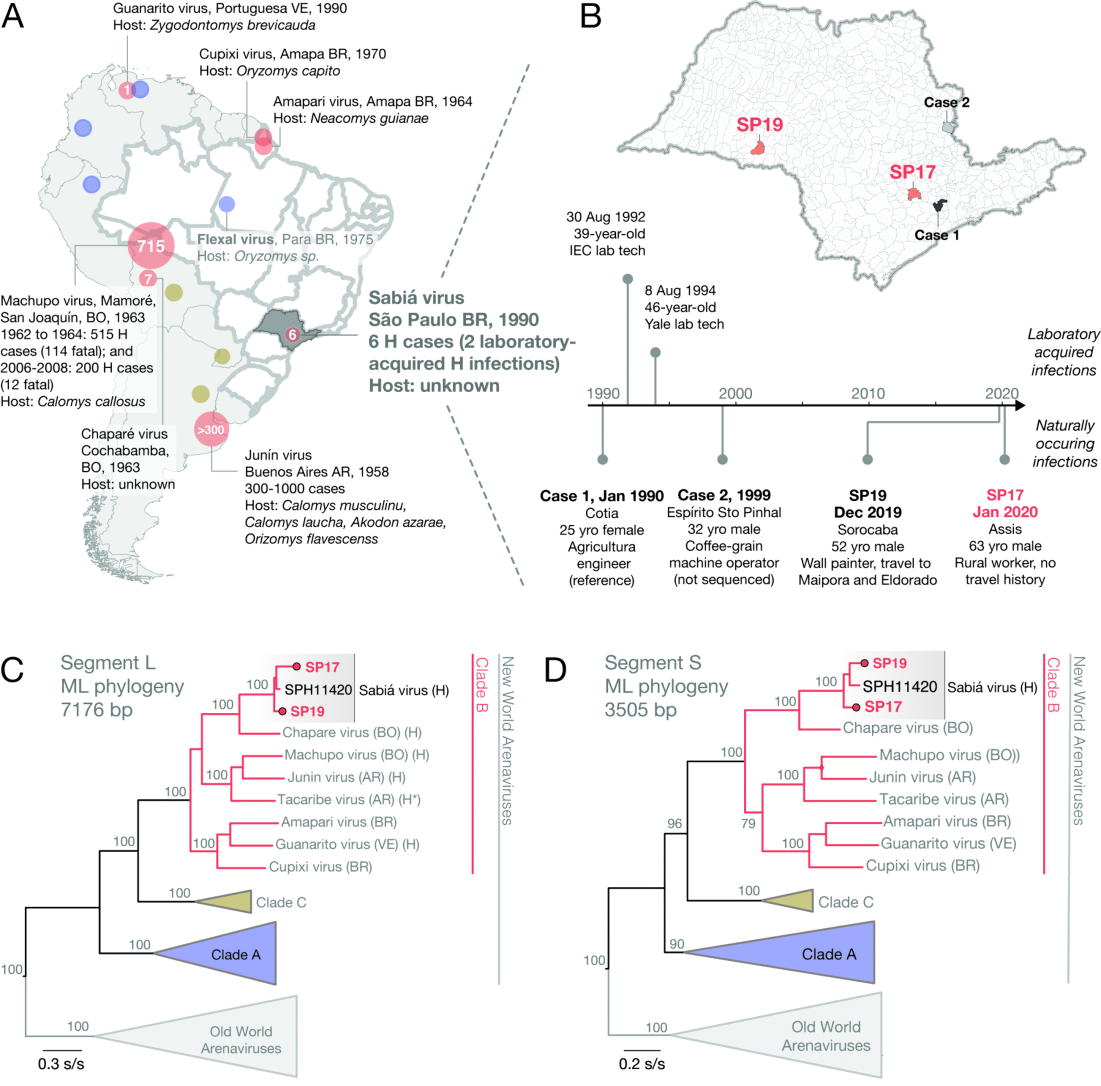
Overview of reported SABV and other human pathogenic arenavirus infections in the Americas. **(A)** Distribution of human pathogenic NWA, including species, year of first detection, state, country, and host information. Circles correspond to reported outbreaks (with case numbers) and are coloured according to NWA clade. São Paulo state, Brazil, is highlighted in dark grey on the map. **(B)** Map of municipalities of São Paulo state, Brazil, with coloured municipalities indicating locations where naturally occurring SABV infections were detected. Distances between cases are: 155 km between Case 1 (1st reported SABV case) and Case 2, 380 km from Case 2 to SP19, and 315 km between SP19 and SP17. Timeline summarizes events related to naturally occurring and laboratory-acquired SABV infections, with SP19 and SP17 highlighted in red. **(C)** and **(D)** ML phylogenetic trees of arenavirus four major clades for L segment and S segment, respectively (Dataset A, https://github.com/CADDE-CENTRE/SABV_Brazil). Tips include virus strain names and countries of identification for clade B NWA viral species. Scale bar = nucleotide substitutions per site (s/s), H = hemorrhagic, L = large, S = small, bp = base pairs, BR = Brazil, AR = Argentina, BO = Bolivia, VE = Venezuela. Maps in panels A and B were created in R version 4.0.1 using the *ggplot2, sf, rnaturalearth, geobr, tmap, lwgeom, units,* and *mapview* packages. Basemap data: Natural Earth (http://www.naturalearthdata.com, public domain) and geobr (based on official IBGE shapefiles, public domain).

Sequencing of seven additional YFV-negative retrospective cases enabled the identification of a previously undetected SABV case, SP19, a 63-year-old male rural-worker from Assis, São Paulo, Brazil, who was admitted to the *Hospital das Clínicas* on December 10, 2019. SP19 reported no travel within two weeks before symptom onset. SP19 experienced a 9-day illness (**Table A in** **S1 Text**). SP19 died on December 12, 2019, with a diagnosis of sepsis in the death certificate (**Fig 1** and **Table A in** **S1 Text**). Both SABV’s positive cases were reported to São Paulo Secretary of Health. Details of infection location, symptom onset, and death dates for SP17, SP19, and previously detected Case 1 (1990) and Case 2 (1999) are shown in **Fig 1** and **Table B in** **S1 Text**. Additional details of clinical manifestations and histopathology were reported elsewhere [10]. Additionally, Patient SP4 was diagnosed with hepatitis A infection, with 99.65% of genome identity with a strain from São Paulo (Accession number: MG049743.1) (**Fig A and Tables A and C in** **S1 Text**). The other five patients were discharged and a viral cause for disease was not identified (**Table A in** **S1 Text**). These findings highlight that multiple viral pathogens may be responsible for hemorrhagic syndromes in YFV-negative cases.

To ensure the reliability of the SABV findings and eliminate the possibility of between-sample or barcode contamination, the two putative-SABV samples were re-sequenced using new flow cells (one per replicate). Samples from SP17 and SP19 yielded 14,423,445 and 6,424,328 reads, respectively, of which 0.12% and 0.002% aligned to the SABV genome reference, resulting in 1,830,030 and 42,622 aligned bases, respectively (**Table C in** **S1 Text**). Genome coverage for SP17 was 100% and 80.1% for segments S and L, respectively. For SP19, the coverage was 57.7% for segment S and 14.2% for segment L. This is probably due to the lower viremia of SABV in SP19, as suggested by the lower fraction of SABV-specific reads in the metagenomic data (0.12% in SP17 versus 0.002% for SP19) and a longer period between symptom onset and sample collection (6 days for SP17 versus 9 days for SP19). Based on a 93.05% amino acid identity at the nucleoprotein (NP) level between SP17 and SP19 and the SABV reference strain SPH114202 (GenBank: NC_006317), our findings support that both genomes belong to SABV, a member of the *Brazilian mammarenavirus* species, consistent with the International Committee on Taxonomy of Viruses (ICTV) criterion for within-species classification in the Arenavirus genus [3]. Furthermore, SP17 and SP19 shared 98.19% amino acid identity at the NP, calculated across the overlapping aligned regions.

At the nucleotide level, the SABV genomes from SP17 and SP19 shared 73.06% identity in segment S and 84.53% in segment L. Compared to the SPH114202 strain (1990), SP17 showed 89.00% and 73.59% nucleotide identity in segments S and L, respectively (**Table D in** **S1 Text**), suggesting that SABV has been circulating in Brazil for decades and that its emergence in humans is not a recent evolutionary event.

**Fig 2:**
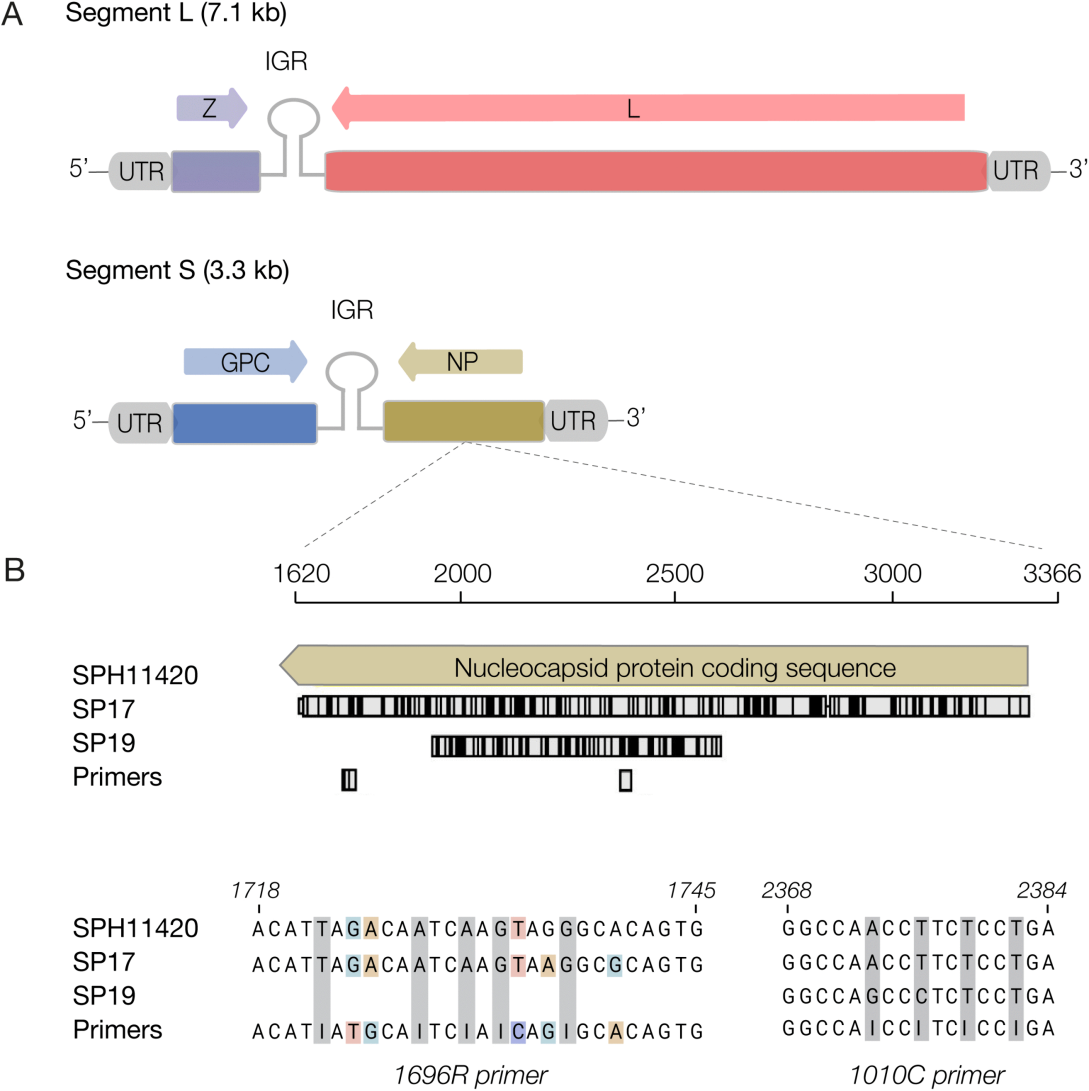
SABV genome structure and primer-binding site mismatches. **(A)** SABV genome structure, featuring an enveloped RNA with a single-stranded ambisense configuration. Segment L encodes the Z matrix protein and the L polymerase protein, while Segment S is responsible for encoding the GP and NP, separated by non-coding intergenic regions (IGR). Linear genomes are flanked by conserved 5’ and 3’ untranslated regions (UTRs) crucial for viral replication and translation processes [13]. **(B)** The upper section displays new strain sequences relative to the SABV reference, primer locations (Bowen et al. [12]), and variant positions in black. The lower part presents primer and SABV sequences at binding sites, with colours indicating primer mismatches and pale grey bars indicating inosine sites. Note: primers are shown in their published 5′ → 3′ orientation. Reverse primers, such as 1010C (5′–TCIGGIGAIGGITGGCC–3′), anneal to the genome by reverse complementarity and therefore may not appear as direct sequence matches when aligned against the 5′–3′ representation of the negative-sense genome. Vs = versus, Z = matrix protein Z, L = RNA-dependent RNA polymerase protein L, GPC = glycoprotein precursor, NP = nucleocapsid protein, IGR = non-coding intergenic regions, UTR = untranslated regions.

Currently, SABV surveillance relies on the Bowen et al [12] protocol designed for the SPH114202 reference strain (1990) which is implemented in some of the Brazilian public health laboratories. We were able to identify two additional nucleotide mismatches to the 1696R primer in the SP17 genome compared to the SPH114202 strain (**Fig 2**), totaling 5 mismatches. Unfortunately, the genomic sequence of SP19 at the 1696R primer site was unavailable for comparison due to insufficient sequencing coverage in this region (**Table D in** **S1 Text**).

Because contemporary SABV genomes show mismatches with existing primers, multiple new assays were developed and evaluated. Based on the SPH114202 and newly generated genomes, we designed six new PCR primer sets and evaluated their performance in detecting contemporary SABV strains (**Tables D–F in** **S1 Text**). Primer sets 1 and 3 were selected for screening based on a combination of their amplification efficiency in preliminary tests and shorter amplicon length, which is advantageous for detecting potentially degraded RNA in field samples. These primer sets were subsequently applied to screen 29 (20 high exposure and nine symptomatic) healthcare workers who had either been in close proximity to SP17 or handled the patient’s blood at *Hospital das Clínicas* and IMT, following the diagnostic framework previously described elsewhere [10]. Next, to detect SABV RNA in SP19, which had lower genome coverage and a reduced proportion of SABV-specific reads, we designed two nested PCR primer sets (**Tables G and H in** **S1 Text**). Our nested PCR assay successfully amplified SABV RNA from the SP19 sample, generating clear DNA bands on agarose gel, despite its low viremia (**Fig D in** **S1 Text**).

**Fig 3:**
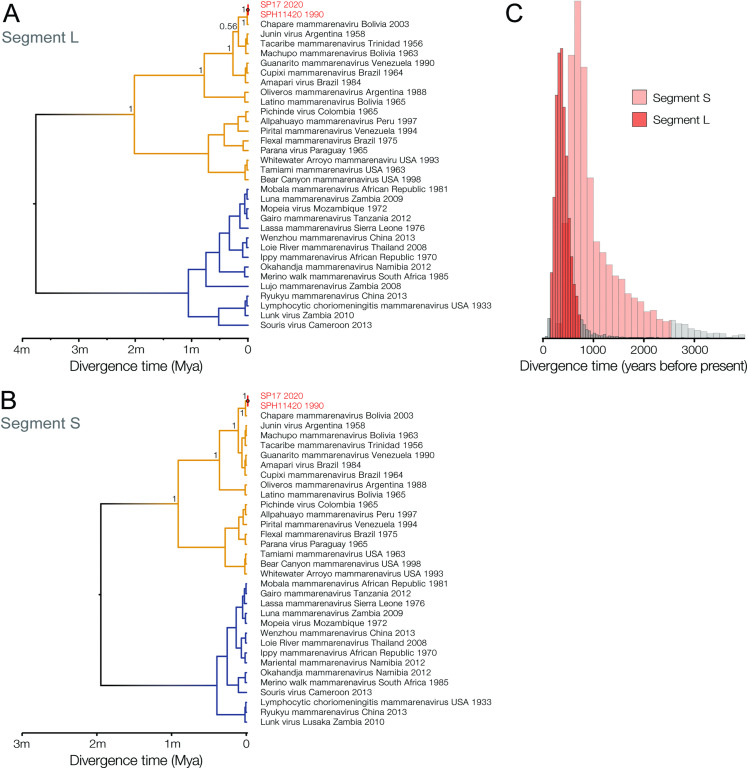
Time-calibrated phylogenies and estimated divergence times for SABV and other arenaviruses. We employ sequence data from SP17 with higher genome coverage for analysis. M = millions, Mya = million years. Inferences were performed for both S **(A)** and L **(B)** segments. **(C)** Posterior distributions for the dates of segments S and L most recent common ancestor.

Maximum likelihood phylogenetic analysis of SABV together with 34 arenavirus species places the SP17, SP19 and SPH114202 strains within Clade B New World arenaviruses (**Figs 1** and 1B **in** **S1 Text**). Both SP17 and SP19 strains clustered with SPH114202 with maximum bootstrap support. We found no evidence of interspecies reassortment or significant genome-wide recombination. These results were further corroborated by Bayesian phylogenetic analyses, which confirmed the SP17, SP19 and SPH114202 cluster with maximum posterior support (**Fig 3**). Using a phylogenetic model that takes into account time-dependency of evolutionary rates, we estimated the most recent common ancestor of SABV around 414 years ago (95% HPD: 142–750) for segment L (**Fig 3A**) and 1,081 years ago (95% HPD: 326–2,539) for segment S (**Fig 3B**). Conservatively, the lower 95% HPD bound suggests SABV has circulated for at least 142 years, with central estimates indicating several centuries. Segment L and S dating estimates overlap significantly, indicating robustness of the findings (**Fig 3C**). SABV shared an ancestral node with Chapare mammarenavirus, dating back to 0.015 million years ago (Mya) (95% HPD: 0.008–0.024 Mya) for segment L and 0.012 Mya (95% HPD: 0.006–0.021 Mya) for segment S. Clade B’s emergence was estimated at approximately 0.17 Mya (95% HPD: 0.13–0.24 Mya) for segment L and 0.11 Mya (95% HPD: 0.06–0.16 Mya) for segment S. NWA and OWA originated around 3.77 Mya (95% HPD: 2.84–4.74 Mya) for segment L and 1.95 Mya (95% HPD: 1.40–2.61 Mya) for segment S.

The SABV GP is divided into stable signal peptide (SSP), GP1 attachment glycoprotein, and GP2 fusion glycoprotein subcomponents, which form a trimer of heterotrimers on the virion surface [14,15]. The GP complex of the new SABV strains share 94% amino acid sequence identity with SPH114202 [2,7] (**Fig E** in **S1 Text**). Our analysis shows that GP2 is more conserved than GP1, consistent with GP2 functional constraints described in other arenaviruses (**Fig E** in **S1 Text**). Specifically, receptor-binding GP1s have a nucleotide identity of 91% with 17 amino acid substitutions, while their GP2s (fusion glycoproteins) have an amino acid identity of 97%, with eight amino acid substitutions. Four amino acid substitutions – F5L, I36V, T51I, and R55K – are in the membrane-inserted SSP (**Fig E** in **S1 Text**). Similar variations have been reported in other arenaviral SSPs substitutions and unlikely to affect GP functionality [16].

**Fig 4:**
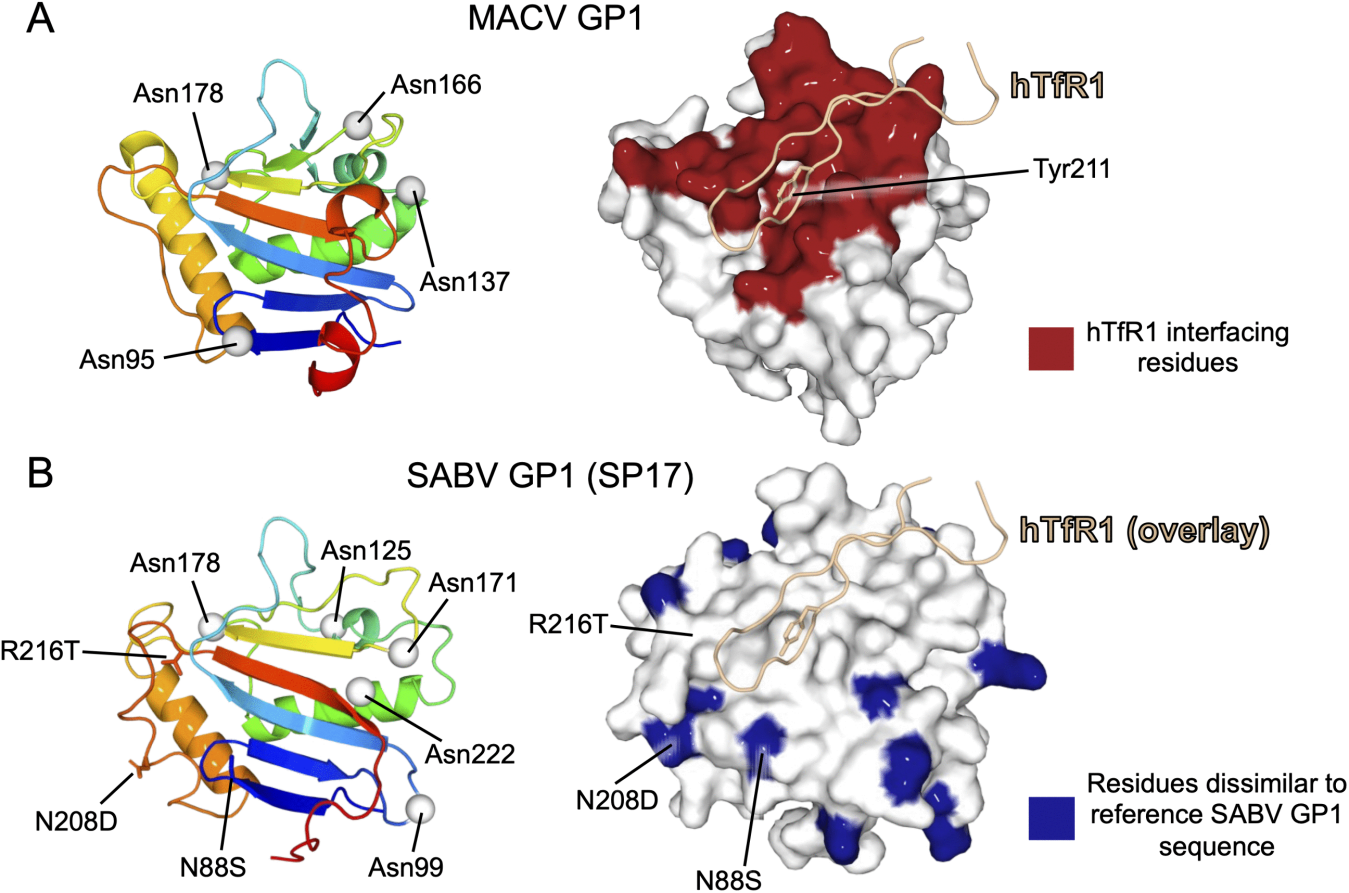
Structural analysis of SABV GP1 mutations. To facilitate comparative analysis of amino acid conservation between the SABV GP1 reference and contemporaneous strains we generated a homology model of SABV GP1 (SP17 strain) using AlphaFold3 [17]. **(A)** Mapping the TfR1-interaction region, an important determinant of zoonotic potential [18], on the surface of the GP1. (Left) Structure of MACV GP1 (PDB no. 3KAS) is shown as a rainbow-colored cartoon from blue (N-) to red (C-terminus) with N-linked glycosylation sites displayed as grey spheres. (Right) Structure of MACV GP1 is shown in white-colored surface representation, with hTfR1-binding residues colored red (determined with PDBePISA [19]. A GP1-interacting chain of hTfR1 is shown as an orange-colored ribbon. The side chain of hTfR1-Tyr211 is shown as a stick. **(B)** Mapping amino acid conservation between SABV GP1 from the reference and contemporaneous strains. (Left) AlphaFold model of the new SABV strain GP1 (SP17), with putative N-linked glycosylation sites displayed as grey spheres. For fair comparison, only residues 80–234 are shown. Substitutions N88S, N208D, and R216T are displayed as sticks. (Right) The model is shown in surface representation and colored white. Amino acid residues dissimilar to the reference SABV GP1 are colored blue. A GP1-interacting chain of hTfR1 (PDB no. 3KAS) is overlaid onto the homology model of SABV GP1 to indicate region of the putative TfR1 binding site. [20].

We generated a homology model of SABV GP1 to examine whether the identified strains exhibit amino acid variation when compared with reference SPH114202 at the putative hTfR1 receptor-binding site (RBS) on the GP1 and thereby reflect different receptor tropism characteristics of the identified viruses (**Fig 4A**). All residues between the SABV GP1 reference and SP17 strains that align with hTfR1-interacting MACV GP1 residues are identical except N208D and R216T (corresponding to Thr216 and Ser224 on MACV GP1, respectively). When mapped onto the SABV GP1 (SP17) homology model, D208 and T216 localize to the periphery of the putative RBS (**Figs 4B**, 4E and 4F in **S1 Text**). It seems plausible that both substitutions are unlikely to have a deleterious effect upon TfR1 binding, whereas N208D will result in minor electrostatic differences within the interface, and the formation of a smaller side-chain in R216T is unlikely to introduce steric hindrance to the interaction. These observations support the hypothesis that a selection pressure exists to maintain TfR1 recognition across SABV strains.

Our mapping analysis also indicates that other identified amino acid differences between SABV GP1 from the 1990 reference strain SPH114202 and the SP17 and SP19 SABV strains localize to putative solvent-accessible surfaces on the GP1 (**Fig 4B**). Given that there is currently no known functional role for these residues, which are placed outside of the putative TfR1-binding region, these substitutions may have arisen out of antigenic pressure. Similarly, the absence of an N-linked glycosylation sequon (N88S) in contemporaneous SABV strains suggests that this putative glycan may not be essential for native functionality or protein folding, and may have evolved in the original SABV sequence to shield protein-specific epitopes from the antibody immune response arising from infection, as has been previously observed for other arenaviruses [20].

## Discussion

Genomic surveillance of recent fatal hemorrhagic cases identified two recent arenavirus infections in São Paulo, Brazil [10,11,21]. Our phylogenetic analysis showed that the viruses sequenced from SP17 and SP19 patient samples belong to the Sabiá virus species, a clade B New World arenavirus. The contemporaneous SABV SP17 strain exhibited variation at the nucleotide and amino acid level, evading detection by existing molecular assays, and our dating phylogenetic analyses indicate that SABV emerged at least over a century ago.

Epidemiological investigations suggest that all naturally occurring infections reported to date have had a history of exposure to rural areas across São Paulo state and resulted in fatal outcomes with diagnoses confirmed post-mortem. Although person-to-person and aerosol transmission are possible transmission routes [5,6], the two SABV cases reported here were linked to forest and rural activities, and no person-to-person transmission was detected among 29 close contacts of patient SP17. This suggests that the two SABV cases reported are most likely the result of distinct introductions from an unsampled reservoir, likely wild rodents [22]. Endemic areas for arenaviruses are expected to expand due to land use changes that increase contacts between reservoirs and humans [23], warranting increased surveillance of potential reservoir hosts. Moreover, three out of four naturally occurring SABV cases detected to date exhibited symptoms during the yellow fever transmission season in São Paulo where nearly half of the deaths attributed to hemorrhagic fever and neuroinvasive disease remain undiagnosed [24]. Prospective serological and molecular testing on patients from Sorocaba/Eldorado and Assis, as well as more broadly on YF-negative cases with hemorrhagic symptoms, could help provide a deeper understanding of SABV epidemiology, including its pathogenicity and transmissibility.

Our analyses of new SABV sequences uncovered substantial genetic diversity of SABV strains (≥10% nucleotide distance) in the NP gene. Synonymous substitutions and within-species diversity align with observations in other arenaviruses, such as Guanarito in Venezuela [25] and Lassa virus in West Africa [26]. Our study also provides a new perspective on an earlier report describing the SP17 strain, which suggested that this strain did not belong to a defined virus species, being characterized as SABV-like mammarenavirus [11]. While experimental verification is needed, mapping sequence conservation onto a model of SABV GP1 showed a high level of sequence conservation at the receptor-binding site and suggested that contemporaneous SABV strains share a TfR1-mediated host-cell entry pathway with the SABV reference strain. Thus, our molecular modeling suggests that contemporaneous SABV strains exhibit conserved species tropism and zoonotic potential characteristics.

Our phylogenetic analyses suggest that New World arenaviruses have circulated for millions of years [22] and show that SABV emerged approximately 1,081 and 414 years ago for the S and L segments, respectively. We show that contemporaneous SABV infections were not detected by existing molecular assays. In São Paulo, nearly half of the deaths attributed to hemorrhagic fever and neuroinvasive disease remain undiagnosed [24]. Therefore, based on current data, it is likely that SABV infections have remained undetected and that the virus has circulated cryptically in São Paulo state, Brazil.

Four NWA virus species, Cupixi, Amapari, Flexal, and Sabiá, have been identified in Brazil. The potential limitations of current molecular diagnostic approaches identified here for SABV underscores the importance of untargeted metagenomic analyses to support outbreak investigations and to better understand NWA epidemiology, evolution and transmission dynamics. Reported SABV infections and other Clade B New World arenavirus infections are associated with high case fatality among detected cases [27,28]; however, this estimate may be inflated towards severe and fatal presentations. It is plausible that mild or subclinical SABV infections occur but remain undetected, as illustrated by the survival of both laboratory-associated cases. Although ribavirin has demonstrated benefit in Lassa fever and has been used empirically in other arenavirus infections [27], evidence supporting its efficacy against Sabiá virus infection is lacking, and clinical outcomes may reflect a spectrum of disease severity rather than antiviral effect. Experimental treatment options for SABV such as ribavirin, combined with the development of rapid point-of-care diagnostic methods, could be critical for patient management.

This study has several limitations. First, due to the unavailability of standardized positive controls (e.g., cloned RNA), we validated the PCR assays using SP17 and SP19 samples, which were genetically characterized through next-generation sequencing. Although this provided practical confirmation in the context of an urgent public health investigation, further validation of the assay’s performance characteristics is required before broader surveillance implementation. Second, the low genome coverage of SP19 limited its use in this study. The absence of full-genome data constrained more comprehensive evolutionary and functional assessments. In addition, other confirmatory methods (e.g., Sanger sequencing) could not be performed due to limited remaining RNA. Virus isolation could not be performed because SABV is classified as a Risk Group 4 pathogen, and Brazil currently lacks the operational BSL-4 laboratory facilities required for such procedures. These constraints further limited the experimental confirmation of infectivity, receptor binding, and viral characteristics for SP19. Third, structural modeling of GP1 was based on limited sequence identity with other arenavirus glycoproteins and thus requires further structural and functional validation. Lastly, the ecological dynamics of SABV and its potential rodent reservoirs remain to be elucidated. Broader genomic, ecological, and serological surveillance efforts are essential to fill these gaps and better understand the risks posed by SABV and other New World arenaviruses in Brazil.

Routine, low-cost, and rapid pathogen-agnostic genomic surveillance is essential to track the circulation of new or divergent viruses and to improve molecular diagnostics. Analysis of YFV-negative genomic data identified this rare, highly lethal SABV as the causative agent. This study ruled out epidemiological links between recent cases and confirmed that close contacts were not infected. It also revealed SABV’s evolutionary history and provided insights into its receptor-binding properties. These findings highlight SABV’s potential for occasional spillover from hidden reservoirs, likely rodents, into human populations and underscore the urgent need to improve SABV diagnostic capacity in Brazil.

## Materials and methods

### Ethics statement

This study was approved by the *Comissão Nacional de Ética em Pesquisa* (CONEP), under protocol number CAAE 82673018.6.1001.0068. All patients signed an informed consent form to participate in the study and provided consent for the future use of their samples (BioBank informed consent).

### Clinical-epidemiological context

The *Hospital das Clínicas* is the main tertiary care hospital associated with the Faculty of Medicine of the University of São Paulo. It offers specialized care to patients with complex medical conditions throughout Brazil. During the YF outbreak in Brazil between 2018 and 2019, the *Hospital das Clínicas* was essential in admitting severe YF cases. Concomitantly, a randomized clinical trial - “SOFFA” study was carried out to assess the effectiveness of sofosbuvir in treating yellow fever [28]. Out of the 67 randomized patients, eight individuals tested negative for YFV RNA in their serum and urine samples, as well as other causes of haemorrhagic fever (HF) (such as dengue virus, hepatovirus A, B, C, Delta, and E, HIV, CMV, EBV, HSV, malaria, leptospirosis, toxoplasmosis, and bacterial septic shock).

### Viral metagenomics

Serum samples from the eight YFV-negative patients were centrifuged at 10,000g for 5 minutes. Then, viral RNA was extracted from the sample supernatant using QIAamp RNA viral extraction kit (Qiagen, Valencia, CA, USA) in a Biosafety Level-3 facility (BSL-3) at the *Instituto de Medicina Tropical* (IMT). Next, extracted RNA was subjected to untargeted metagenomic sequencing using the SMART-9N protocol [29]. Libraries were prepared using the SQK-LSK109 ligation sequencing kit and sequenced on the MinION platform (Oxford Nanopore Technologies, ONT, UK). Additional protocol details are provided in S1 Text.

Each sample was subjected to two full technical replicates before sequencing on separate and previously unused R9.4.1 flow cells (ONT, UK), with a negative control added in each reaction. Raw files were demultiplexed using Guppy in High Accuracy mode (ONT, UK). Fastq files were taxonomically classified using the standard Kraken2 database [30], and the results were visualized using the pavian tool [31]. Identified reads were mapped against the SABV reference genome (S and L segments, accession numbers AY358026 and U41071) with minimap2 version 2·17-r941 with parameters ‘-a’ (SAM output) and ‘-x map-ont’ (nanopore mapping mode), ‘-w1’ and ‘-k11’ (increased sensitivity) [32].

Genome statistics were assessed with Samtools [33] and the Tablet viewer [34]. To recover consensus sequences from the S and L genomic fragments, we called variants detected with Medaka [35] for regions of the genome covered with at least 5 reads. Consensus sequences were subjected to BLAST searches against the NCBI viral protein and nucleotide sequence databases, and the percent identity were determined between the new SABV genomes and available SABV reference sequences [36]. The sequences, along with available information, were immediately shared with the São Paulo Secretary of Health and included in a report to support further research and public health initiatives [21]. The genome sequences of sample SP17 have been submitted to GenBank under accession numbers PX308626 and PX308627, while the partial genome sequences of sample SP19 are available at https://github.com/CADDE-CENTRE/SABV_Brazil.

### Phylogenetic analyses

To investigate SABV diversity within the *Arenaviridae* family, phylogenetic analyses were conducted for the L and S genome segments. Reference datasets from the International Committee on Taxonomy of Viruses (ICTV) included a nucleotide (nt) and amino acid (aa) genome sequence per viral species from Clades A, B, and C of the New World arenavirus (NWA) and the Old World arenavirus (OWA) complexes (n = 36, dataset A - https://github.com/CADDE-CENTRE/SABV_Brazil). Phylogenies were estimated using GTR nucleotide substitution model and a blosum62 aa exchange matrix for nt and aa-based datasets, respectively, in PhyML [37] using Seaview [38]. Branch support was assessed using an approximate likelihood ratio test [39].

To confirm the phylogenetic placement of the newly generated SABV sequences, we downloaded 6,143 coding sequences of the Mammarenavirus genus from the NCBI Virus platform (https://ncbi.nlm.nih.gov/labs/virus/vssi/#/virus). We filtered sequences for the NP, GP, Z, and RdRp ORFs based on length thresholds, set at 70% of the corresponding protein length in the Lassa mammarenavirus (LASV) RefSeq genome (segment S: NC_004296, segment L: NC_004297). Sequences lacking collection dates or containing stop codons were removed, yielding reference datasets of 862 (NP), 891 (GP), 708 (Z), and 755 (RdRp) nucleotide sequences, respectively (Dataset B, available at https://github.com/CADDE-CENTRE/SABV_Brazil). The final datasets were combined with the newly generated SABV sequences, and phylogenetic trees were inferred using IQ-TREE v2 [40] with the GTR + F + I + G4 nucleotide substitution model and 1,000 maximum-likelihood bootstrap replicates.

To infer the evolutionary history of SABV, dated phylogenetic trees were estimated from the nucleotide dataset A in BEAST v1.10.4 [41] using the BEAGLE v3 library [41]. We applied a time-dependent rate (TDR) model [42] on the deep SABV history, with an informative prior on the modern rate (a normal distribution with mean -6.7 and standard deviation of 0.25 on the log rate) based on published LASV virus evolutionary rates [43] and an informative prior on the TDR decline rate (normal with mean -6.21 and standard deviation of 0.035) based on previous TDR estimates [42]. Furthermore, we specified a GTR + Γ substitution model and a Yule tree prior across the S and L segments. Posterior distributions of the time of the most recent common ancestor of SABV strains from the S and L dated phylogenetic distributions were obtained using TreeStat v.10.4 [41]. Potential recombination events of the NWA were investigated from dataset A, using all available methods in RDP4 [44].

### Homology modeling and structure-based mapping analysis of SABV GP1

Amino acid sequences of SABV envelope glycoprotein GP1 (F59 − S250; Sample 17 (SP17)) were submitted to AlphaFold 3 server [17], with no additional customisation. SABV GP1 model with a predicted template modelling (pTM) score of 0.73 was chosen and used for structural-based mapping analysis, performed in PyMol - https://pymol.org. Putative asparagine (N)-linked glycosylation sites were predicted using NetNGlyc [45].

### Development of a PCR and sensitive nested-cPCR assays

To investigate whether primer sets from current assays would detect the new SABV strains, we evaluated the performance of six new RT-PCR primer pairs (**Table E in** **S1 Text**). These primers were designed based on the 1990 reference genome of SABV and sequences newly obtained in this study. We selected conserved regions within the NP gene to ensure assay sensitivity and specificity. Degenerate bases were included in regions with sequence variation to improve compatibility with circulating strains. Briefly, the extracted RNA from SP17 and SP19 patient samples, and a negative control was converted into cDNA using Random Hexamers (Thermo Fisher Scientific, USA) and ProtoScript II First Strand cDNA Synthesis Kit (New England Biolabs, UK) following manufacturer’s instructions. The RT-PCR reactions were prepared using 5 μl Q5 reaction buffer (New England BioLabs, USA), 0·5 μl 10 μM dNTP, 1·25 μl of the forward primer, and 1·25 μl of the reverse primer (variable, according to the primer set, 10 μM), 14·25 μl Nuclease-free water (NFW), 0·25 μl Q5 DNA polymerase, and 2·5 μl of cDNA (**Tables F and G in** **S1 Text**). PCR cycling conditions were: 98°C for 45 sec, followed by 30 cycles of 98°C for 15 sec, Tm variable for 15 sec, and 65°C for 5 min and a final step of 65°C for 10 min, changing the Tm according to the set of primers used (**Tables E–G in** **S1 Text**). A gel was prepared using the E-gel EX Agarose 2% (Thermo Fisher Scientific, USA). To load the PCR products, 5 μl of the products were mixed with 15 μl of NFW and 20 μl of E-gel Sizing DNA leader (Thermo Fisher Scientific, USA) was used in the first band. The gel was placed into the E-gel equipment (Thermo Fisher Scientific, USA) and the run was performed until the bands were distinguishable by transillumination.

We next designed two nested-cPCR primer sets in an attempt to detect low viremia cases (**Table H in** **S1 Text**). The nested-cPCR reactions were prepared using 5 μl Q5 reaction buffer (New England BioLabs, USA), 0·5 μl 10 μM dNTP, 1 μl of the forward primer, and 1 μl of the reverse primer (variable, according to the primer set, 10 μM), 14·75 μl Nuclease-free water (NFW), 0·25 μl Q5 DNA polymerase, and 2·5 μl of cDNA from SP19 (**Tables H–J in** **S1 Text**). PCR cycling conditions were: 98°C for 45 sec, followed by 30 cycles of 98°C for 15 sec, Tm variable for 15 sec, and 65°C for 5 min and a final step of 65°C for 10 min. This reaction was done twice, once for the outer PCR reaction, and then for the inner PCR reaction, changing the Tm according to the set of primers used (**Tables H–J in** **S1 Text**). 5 μl of the products were mixed with 15 μl of NFW and 20 μl of E-gel Sizing DNA leader were loaded to the bands visualization (Thermo Fisher Scientific, USA). The gel was placed into the E-gel equipment (Thermo Fisher Scientific, USA) and the run was performed until the bands were distinguishable by transillumination.

Following validation using SABV-positive clinical samples, selected primer sets were applied to screen 29 high-risk healthcare worker contacts, as part of the contact investigation previously described by Nastri et al. [10]. Detailed laboratory protocols used in this study have been deposited in protocols.io and are publicly available at: https://dx.doi.org/10.17504/protocols.io.4r3l2z293l1y/v1.

## Supporting information

S1 Text Fig A. Pavian plots of Kraken2 database matches. Pavian diagrams of Kraken 2 report results obtained from SP4. The width of the flow is proportional to the number of reads. The number above each node is the number of k-mer hits. A rank code, indicating domain (D), phylum (P), family (F), genus (G), or species (S) was used. **Fig B.** Phylogenetic trees for Z, L, GP and NP SABV proteins. Maximum Likelihood phylogenetic trees of arenavirus species based on nucleotide (nt) alignments of Dataset B (available at https://github.com/CADDE-CENTRE/Sabia). The trees show two groups of arenaviruses, the Old World arenaviruses and the New World arenaviruses. The scale bar represents the units of nucleotide substitutions per site. SABV strains are distinctly highlighted with a box colored in a green gradient. The green tips indicate the patient’s samples SP17 and SP19 described in this study. GPC = glycoprotein complex, Z = zinc-finger-like protein NP = nucleoprotein, RdRP = RNA-dependent RNA polymerase also known as L protein. **Fig. C.** RT-PCR validation for different SABV primer sets. Left panel: the bands highlighted by a circle refer to the primer sets 1 (350 bp), 2 (200 bp), and 3 (121 bp) respectively; Right panel: no band was detected using primer set 4 and the bands highlighted by a circle refer to the primer sets 5 (723 bp) and 6 (200 bp), respectively. (NC: Negative control; bp: base pairs; Sample 17 = SP17; Sample 19 = SP19). **Fig D.** Nested cPCR validation for SABV. The bands highlighted by a circle refer to primer sets 1 (200 bp) and 2 (112 bp). (NC: Negative control; bp: base pairs; Sample 19 = SP19). **Fig E.** Sequence alignments of SABV GP1. (Top) An amino acid sequence alignment of the SABV GP complex from SP17 and reference SPH114202 strains. A sequence consensus line is displayed below the alignment. A highly conserved residue appears in high-consensus red color and as an uppercase letter in the consensus line while a weakly conserved residue is colored blue and as a lowercase letter. A position with no conserved residue is represented by a dot in the consensus line. Symbol! represents I and V, $ for L and M, % for F and Y, # for N, D, Q, and E. (Bottom) An amino acid sequence alignment of MACV GP1, SABV SPH114202 GP1, and SABV GP1 from SP17. The sequence consensus and color scheme are as the top panel. Both alignments were plotted using MultAlin (13). **Fig F.** Analysis of MACV GP1 residues that interact with hTfR1. The structure of MACV GP1 in complex with hTfR1 (PDB no. 3KAS) was submitted to PDBePISA to analyze the interaction interface. The calculated results show accessible/buried surface area (ASA/BSA; Å2), solvation energy effect (ΔiG; kcal/mol), and intermolecular interactions (HSDC stands for hydrogen bond, salt bridge, disulphide bond and covalent link). Buried area percentage is represented by vertical bars (one bar per 10%). Residues on light blue background are solvent accessible residues while those on a darker blue shade are inaccessible residues. Only MACV GP1 (“Structure 2”) residues 86–241 are shown here. **Table A.** Epidemiological characteristics of eight YFV-negative cases. ID = identity; Patients were also previously tested negative for the viruses dengue, hepatovirus A, B, C Delta, and E, HIV, CMV, EBV, and HSV, as well as malaria, leptospirosis, toxoplasmosis, and bacterial septic shock. **Table B.** Epidemiological characteristics of all reported SABV cases. YFV = Yellow fever virus; USA = United States of America; NA = natural acquired. Cases from Dec 2019 were most likely infected in Assis (420 km from Cotia) and Sorocaba/Eldorado (65/210 km from Cotia). Distance between Assis and Sorocaba or Eldorado is 356/430 km. All patients had a fatal clinical outcome. No genomic report exists of strain Case 2 (SPH185338). **Table C.** Sequencing and alignment statistics for the SABV and HAV cases analyzed in this report, including sequencing coverage statistics. **Table D.** Nucleotide and amino acid identity among SABV strains. **Table E.** SABV primer sets used for validation of an RT-PCR assay. Primers were designed based on the 1990 reference sequence of SABV and newly generated genomes from this study. Conserved regions of the nucleoprotein (NP) gene were selected as the amplification targets due to their diagnostic relevance and sequence conservation across SABV strains. **Table F.** Reagents and conditions for the SABV RT-PCR protocol. **Table G.** PCR cycling conditions for the SABV RT-PCR detection protocol. **Table H.** Primer sequences used in the nested-cPCR reactions. **Table I.** Nested cPCR reagents and conditions for the SABV detection protocols. Note that the reaction is done twice, once for the outer PCR reaction, and then for the inner PCR reaction. **Table J.** PCR cycling conditions for the SABV nested cPCR detection protocol. Note that the reaction is done twice, once for the outer PCR reaction, and then for the inner PCR reaction.
(DOCX)
