# Supplementary material for: Genomic characterization of Sabiá virus in Brazil, 2019–2020: Implications for diagnostics, virus evolution, and receptor binding
Source: PLoS Negl Trop Dis. 2026 Feb 20;20(2):e0014008. doi: 10.1371/journal.pntd.0014008 (PMC12945313; doi:10.1371/journal.pntd.0014008)
Supplement: S1 Text — Pavian plots of Kraken2 database matches. Pavian diagrams of Kraken 2 report results obtained from SP4. The width of the flow is proportional to the number of reads. The number above each node is the number of k-mer hits. A rank code, indicating domain (D), phylum (P), family (F), genus (G), or species (S) was used. Fig B. Phylogenetic trees for Z, L, GP and NP SABV proteins. Maximum Likelihood phylogenetic trees of arenavirus species based on nucleotide (nt) alignments of Dataset B (available at https://github.com/CADDE-CENTRE/Sabia). The trees show two groups of arenaviruses, the Old World arenaviruses and the New World arenaviruses. The scale bar represents the units of nucleotide substitutions per site. SABV strains are distinctly highlighted with a box colored in a green gradient. The green tips indicate the patient’s samples SP17 and SP19 described in this study. GPC = glycoprotein complex, Z = zinc-finger-like protein NP = nucleoprotein, RdRP = RNA-dependent RNA polymerase also known as L protein. Fig. C. RT-PCR validation for different SABV primer sets. Left panel: the bands highlighted by a circle refer to the primer sets 1 (350 bp), 2 (200 bp), and 3 (121 bp) respectively; Right panel: no band was detected using primer set 4 and the bands highlighted by a circle refer to the primer sets 5 (723 bp) and 6 (200 bp), respectively. (NC: Negative control; bp: base pairs; Sample 17 = SP17; Sample 19 = SP19). Fig D. Nested cPCR validation for SABV. The bands highlighted by a circle refer to primer sets 1 (200 bp) and 2 (112 bp). (NC: Negative control; bp: base pairs; Sample 19 = SP19). Fig E. Sequence alignments of SABV GP1. (Top) An amino acid sequence alignment of the SABV GP complex from SP17 and reference SPH114202 strains. A sequence consensus line is displayed below the alignment. A highly conserved residue appears in high-consensus red color and as an uppercase letter in the consensus line while a weakly conserved residue is colored blue and as a lowercase [file pntd.0014008.s001.docx]

**S1 Text – Supporting Information**

**Genomic characterization of Sabiá virus in Brazil: implications for diagnostics, virus evolution, and receptor binding**

**List of Contents**

|  | **Page** |
| --- | --- |
| **Materials and Methods** (Supporting Information) | **1** |
| **Fig A.** Pavian plots of Kraken2 database matches. | **4** |
| **Fig B.** Phylogenetic trees for Z, L, GP and NP SABV proteins. | **5** |
| **Fig C.** RT-PCR validation for different SABV primer sets. | **6** |
| **Fig D.** Nested cPCR validation for SABV. | **7** |
| **Fig E.** Sequence alignments of SABV GP1. | **8** |
| **Fig F.** Analysis of MACV GP1 residues that interact with hTfR1. | **9** |
| **Table A.** Epidemiological characteristics of eight YFV-negative cases. | **10** |
| **Table B.** Epidemiological characteristics of all reported SABV cases. | **11** |
| **Table C.** Sequencing and alignment statistics for the SABV and HAV cases analyzed in this report, including sequencing coverage statistics. | **12** |
| **Table D.** Nucleotide and amino acid identity among SABV strains. | **13** |
| **Table E.** SABV primer sets used for validation of an RT-PCR assay. | **14** |
| **Table F.** Reagents and conditions for the SABV RT-PCR protocol. | **15** |
| **Table G.** PCR cycling conditions for the SABV RT-PCR detection protocol. | **16** |
| **Table H.** Primer sequences used in the nested-cPCR reactions. | **17** |
| **Table I.** Nested cPCR reagents and conditions for the SABV detection protocols. | **18** |
| **Table J**. PCR cycling conditions for the SABV nested cPCR detection protocol. | **19** |
| **References** | **20** |

**Materials and Methods**

**Metagenomics sequencing and bioinformatics**

Plasma samples from the eight YFV-negative patients were processed using the SMART-9N metagenomic protocol [(1)](https://paperpile.com/c/EQ6D8X/We342). Briefly, patients’ samples were collected in EDTA tubes. Plasma was separated into two aliquots and centrifuged at 10,000 g for 5 minutes. RNA was extracted from 200 μL of the sample supernatant using QIAamp Viral RNA Mini Kit (Qiagen, Germany) in a BSL-3 at the Institute of Tropical Medicine, Faculdade de Medicina, Universidade de São Paulo (IMT-FMUSP), Brazil, according to the manufacturer's instructions and eluted in 50 μl of elution buffer. The extracted RNA was treated using TURBO DNase (Thermo Fisher Scientific, USA) at 37°C for 30 min to remove residual DNA before being cleaned up and concentrated to 10 μl using Zymo RNA clean & concentrator-5 (Zymo Research, USA).

Sequencing was conducted using the MinION platform from Oxford Nanopore Technologies, UK (ONT, UK) using 50 ng of each amplified cDNA, barcoded, and pooled in an equimolar fashion using the EXP-NBD104 (1–12) and EXP-NBD114 (13–24) Native Barcoding Kits (ONT, UK). Sequencing libraries were generated using the SQK-LSK109 Kit (ONT, UK) and sequenced using MinKNOW 1·15·1 with the standard 48-hours run script. Each sample was subjected to two full technical replicates (RNA extraction, cDNA synthesis, PCR, library generation) before running on separate, fresh ONT flow cells (one per replicate). Raw files were demultiplexed using Guppy in High Accuracy (HAC) mode (ONT, UK). Fastq files for each sample were taxonomically classified using the standard Kraken2 database [(2)](https://paperpile.com/c/EQ6D8X/6HeJD), and the results were visualized using the pavian tool [(3)](https://paperpile.com/c/EQ6D8X/xwCln). After viral identification, reads were mapped against the SABV reference genome (S and L segments, Accession numbers NC_006317·1 and NC_00631·1) with minimap2 version 2·17-r941 with parameters ‘-a’ (SAM output) and ‘-x map-ont’ (nanopore mapping mode), ‘-w1’ and ‘-k11’ (increased sensitivity) [(4)](https://paperpile.com/c/EQ6D8X/9v6L4).

Genome statistics were obtained from Samtools [(5)](https://paperpile.com/c/EQ6D8X/4CiLT) and the [Tablet viewer](https://ics.hutton.ac.uk/tablet/) [(6)](https://paperpile.com/c/EQ6D8X/5QAbb). To recover consensus sequences from the S and L genomic fragments, we called variants detected with [Medaka](https://nanoporetech.github.io/medaka/) [(7)](https://paperpile.com/c/EQ6D8X/1dYg6) for regions of the genome covered with at least 5 reads. Consensus sequences were subjected to BLAST searches against the NCBI viral protein and nucleotide sequence databases and the percent identity were determined between the new SABV genomes and available SABV reference sequences, masking and excluding regions without coverage [(8)](https://paperpile.com/c/EQ6D8X/O6tY). SP17 and SP19 sequences have submitted to GenBank under Accession numbers PX308626 and PX308627. In addition, partial genome assemblies are publicly available in our GitHub repository: <https://github.com/CADDE-CENTRE/SABV_Brazil>.

**Phylogenetic analysis**

To confirm phylogenetic placement of the SABV novel sequences, we downloaded 6,143 coding sequences from the Mammarenavirus genus from the NCBI virus platform [(9)](https://paperpile.com/c/EQ6D8X/jI9BW). Sequences for the ORFs nucleoprotein, glycoprotein, z-protein and RdRp were filtered by length, with this threshold corresponding to 70% of the length of each protein in the Lassa mammarenavirus (LASV) RefSeq genome (segment S: NC_004296, segment L: NC_004297). Sequences lacking collection dates or presenting stop codons were removed. This process yielded reference datasets containing 862 (nucleoprotein), 891 (glycoprotein), 708 (Z protein) and 755 (RdRP) nt sequences respectively (Dataset B, available at https://github.com/CADDE-CENTRE/Sabia). The final databases were combined with the recently SABV generated sequences, aligned using MAFFT (version 7) [(10)](https://paperpile.com/c/EQ6D8X/wEcA2), and trimmed to their coding regions. Sequences were inspected using AliView (version 1.26) [(11)](https://paperpile.com/c/EQ6D8X/mutZR), followed by the estimation of the maximum-likelihood phylogenetic tree in IQTree V.2 [(12)](https://paperpile.com/c/EQ6D8X/aozfj) using a GTR+F+I+G4 nucleotide substitution model, with 1000 full ML bootstrap replicates.

**Development of SABV RT-PCR and nested-cPCR assays**

The extracted RNA from SP17 and SP19 patient samples, and a negative control was converted into cDNA using Random Hexamers (Thermo Fisher Scientific, USA) and ProtoScript II First Strand cDNA Synthesis Kit (New England Biolabs, UK) following manufacturer’s instructions. The RT-PCR reactions were prepared using 5 μl Q5 reaction buffer (New England BioLabs, USA), 0·5 μl 10 μM dNTP, 1·25 μl of the forward primer, and 1·25 μl of the reverse primer (variable, according to the primer set, 10 μM), 14·25 μl Nuclease-free water (NFW), 0·25 μl Q5 DNA polymerase, and 2·5 μl of cDNA (**Tables S6, and S7**). PCR cycling conditions were: 98°C for 45 sec, followed by 30 cycles of 98°C for 15 sec, Tm variable for 15 sec, and 65°C for 5 min and a final step of 65°C for 10 min, changing the Tm according to the set of primers used (**Tables S5-S7**). A gel was prepared using the E-gel EX Agarose 2% (Thermo Fisher Scientific, USA). To load the PCR products, 5 μl of the products were mixed with 15 μl of NFW and 20 μl of E-gel Sizing DNA leader (Thermo Fisher Scientific, USA) was used in the first band. The gel was placed into the E-gel equipment (Thermo Fisher Scientific, USA) and the run was performed until the bands were distinguishable by transillumination.

We next designed two nested-cPCR primer sets to detect low viremia cases (**Table S8**). The nested-cPCR reactions were prepared using 5 μl Q5 reaction buffer (New England BioLabs, USA), 0·5 μl 10 μM dNTP, 1 μl of the forward primer, and 1 μl of the reverse primer (variable, according to the primer set, 10 μM), 14·75 μl Nuclease-free water (NFW), 0·25 μl Q5 DNA polymerase, and 2·5 μl of cDNA from SP19 (**Tables S8-S10**). PCR cycling conditions were: 98°C for 45 sec, followed by 30 cycles of 98°C for 15 sec, Tm variable for 15 sec, and 65°C for 5 min and a final step of 65°C for 10 min. This reaction was done twice, once for the outer PCR reaction, and then for the inner PCR reaction, changing the Tm according to the set of primers used (**Tables S8-S10**). 5 μl of the products were mixed with 15 μl of NFW and 20 μl of E-gel Sizing DNA leader were loaded to the bands visualization (Thermo Fisher Scientific, USA). The gel was placed into the E-gel equipment (Thermo Fisher Scientific, USA) and the run was performed until the bands were distinguishable by transillumination.

After validation, 117 healthcare workers who were exposed to case SP17 were tested with the RT-PCR, including 21 doctors, 57 nursing professionals, four physiotherapists, three dentists, 21 cleaning workers, and ten laboratory professionals.

**Supplementary Information Figures**


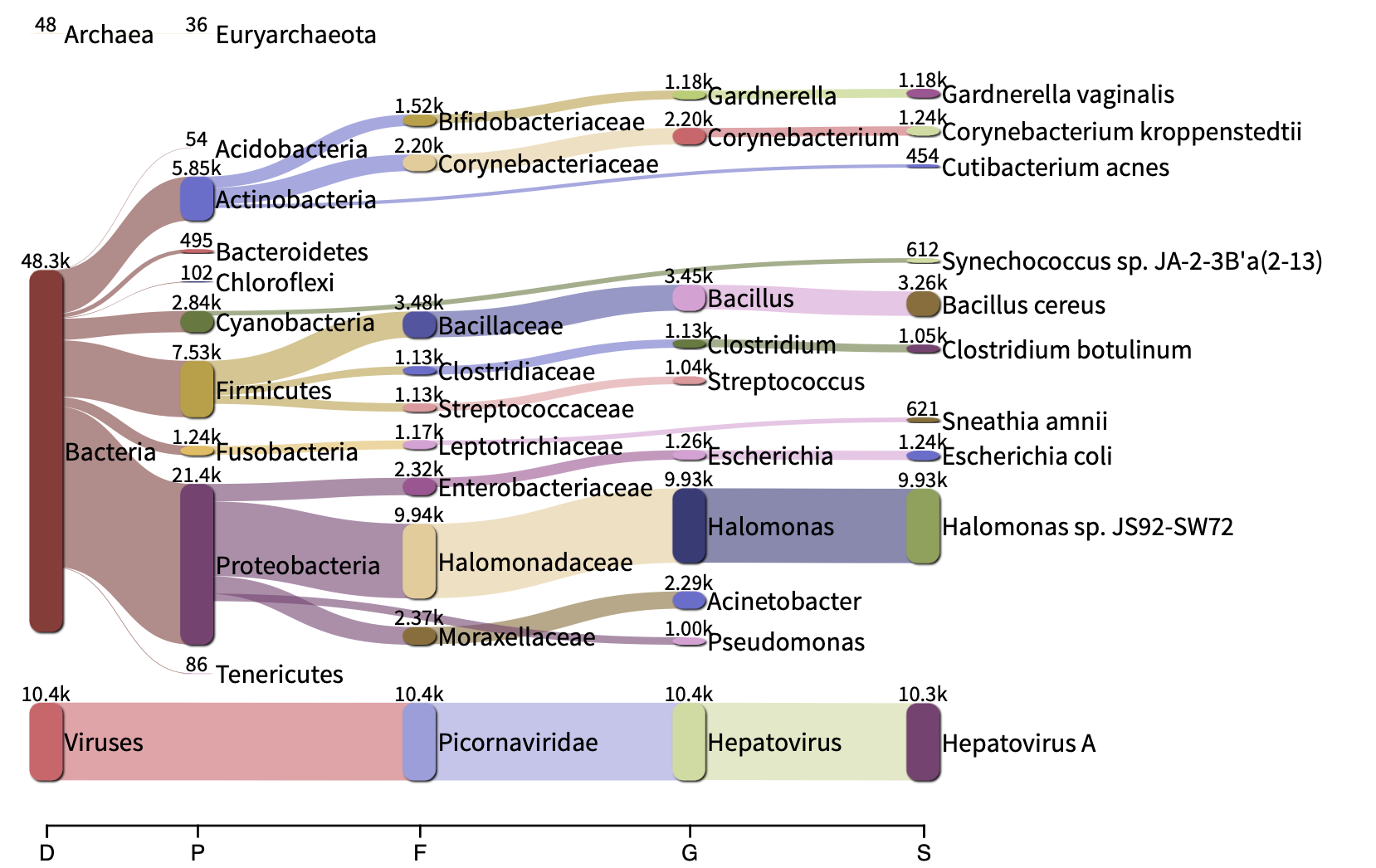


**Fig A. Pavian plots of Kraken2 database matches.** Pavian diagrams of Kraken 2 report results obtained from SP4. The width of the flow is proportional to the number of reads. The number above each node is the number of k-mer hits. A rank code, indicating domain (D), phylum (P), family (F), genus (G), or species (S) was used.

**
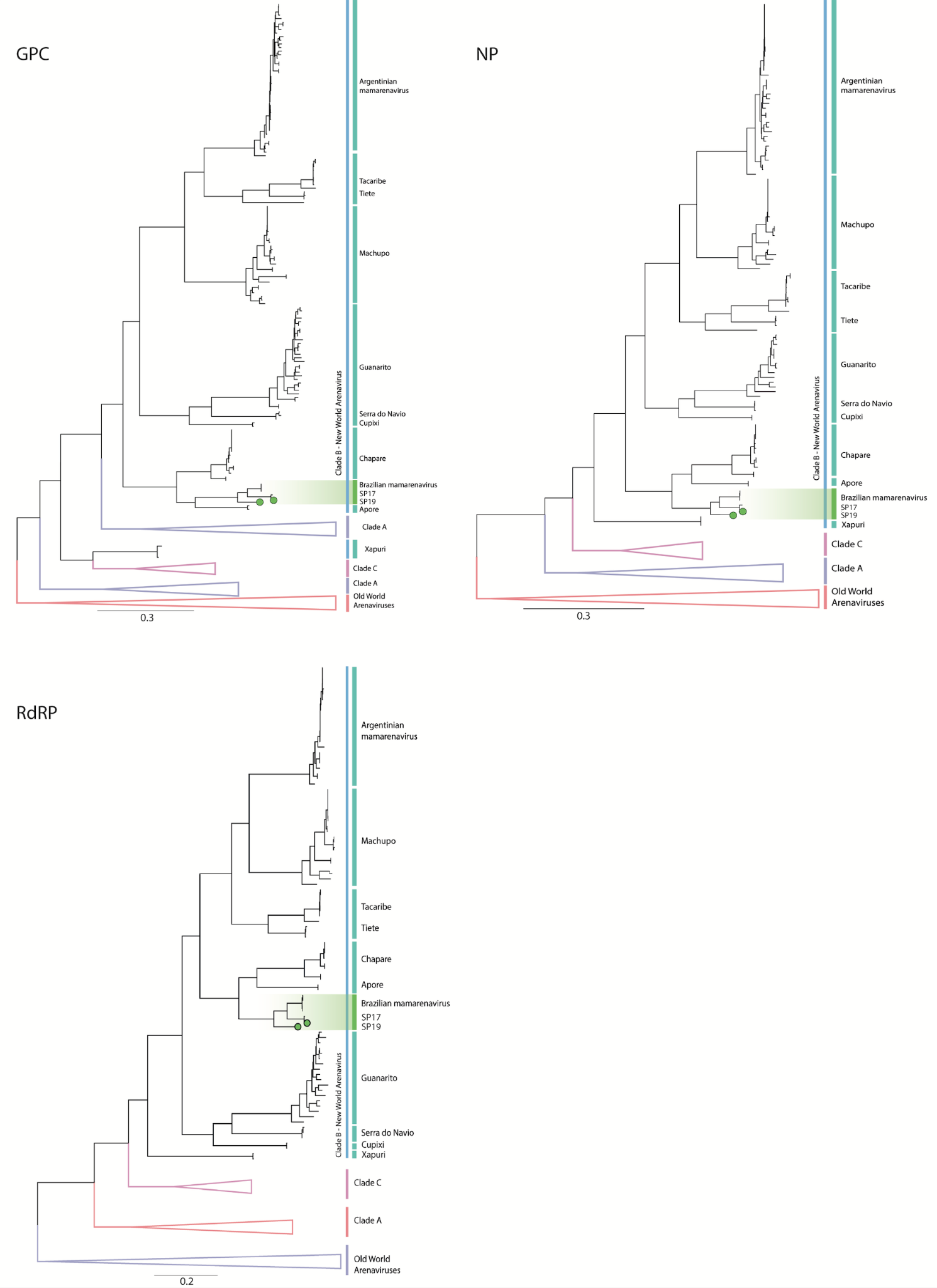
**

**Fig B. Phylogenetic trees for Z, L, GP and NP SABV proteins**. Maximum Likelihood phylogenetic trees of arenavirus species based on nucleotide (nt) alignments of Dataset B (available at https://github.com/CADDE-CENTRE/Sabia). The trees show two groups of arenaviruses, the Old-World arenaviruses and the New World arenaviruses. The scale bar represents the units of nucleotide substitutions per site. SABV strains are distinctly highlighted with a box colored in a green gradient. The green tips indicate the patient's samples SP17 and SP19 described in this study. GPC = glycoprotein complex, Z = zinc-finger-like protein NP = nucleoprotein, RdRP = RNA-dependent RNA polymerase also known as L protein.


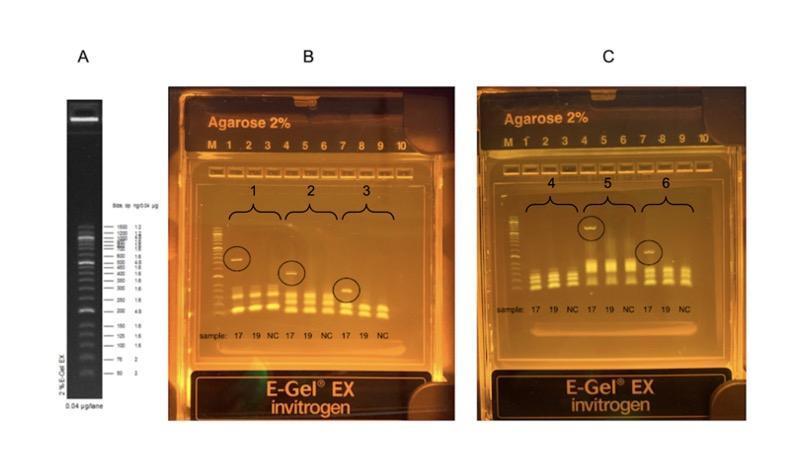


**Fig C. RT-PCR validation for different SABV primer sets.** Left panel: the bands highlighted by a circle refer to the primer sets 1 (350bp), 2 (200bp), and 3 (121bp) respectively; Right panel: no band was detected using primer set 4 and the bands highlighted by a circle refer to the primer sets 5 (723bp) and 6 (200bp), respectively. (*NC*: Negative control; *bp: base pairs; Sample 17 = SP17*; *Sample 19 = SP19*).


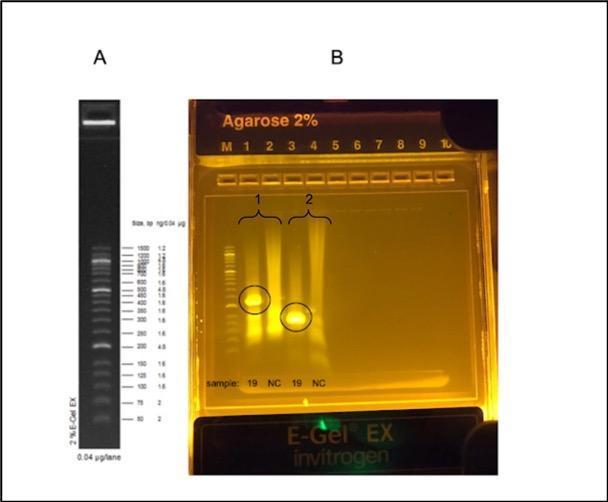


**Fig D. Nested cPCR validation for SABV.** The bands highlighted by a circle refer to primer sets 1 (200bp) and 2 (112bp). (*NC*: Negative control; *bp: base pairs*; *Sample 19 = SP19*).

**
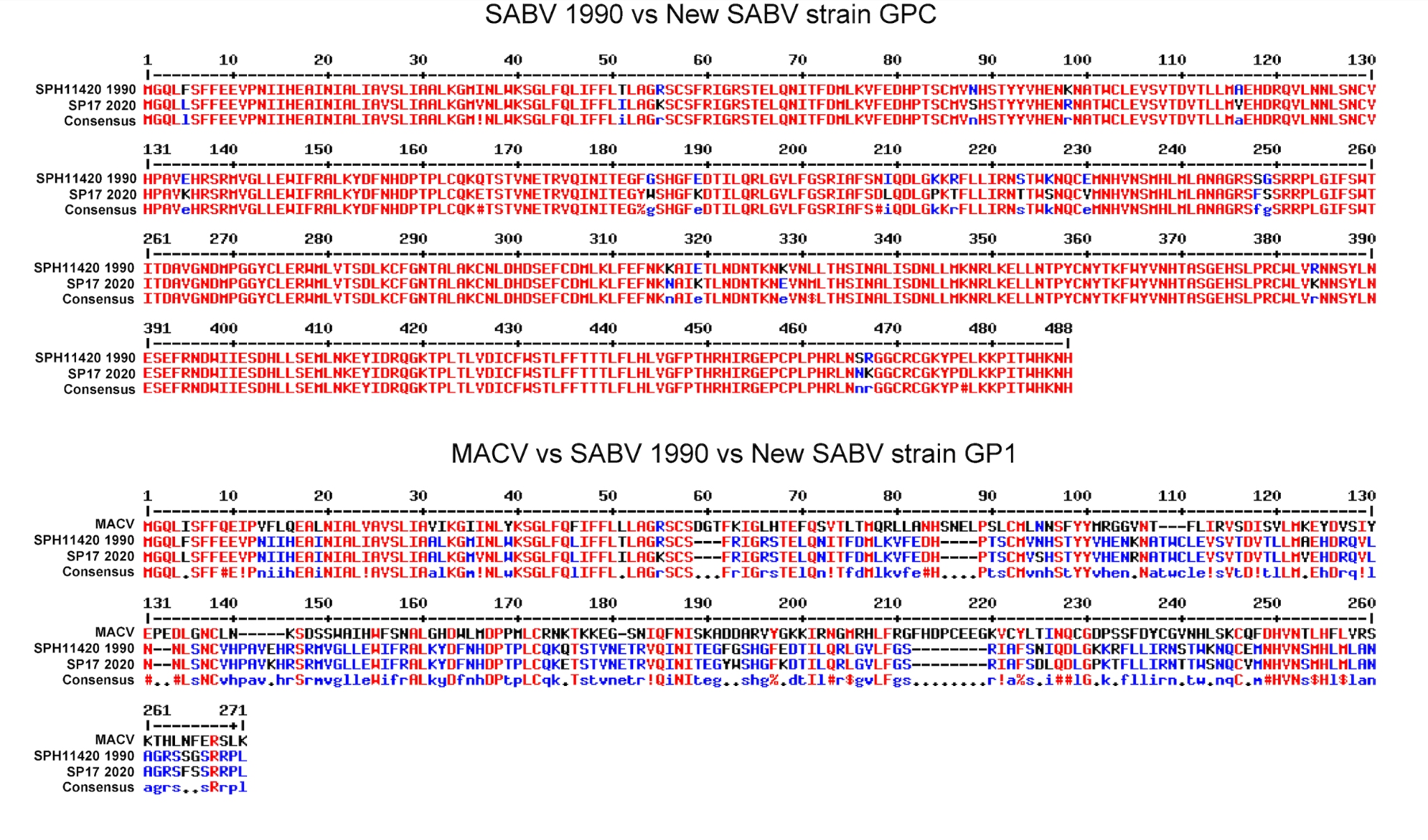
**

**Fig E. Sequence alignments of SABV GP1.** (*Top*) An amino acid sequence alignment of the SABV GP complex from SP17 and reference SPH114202 strains. A sequence consensus line is displayed below the alignment. A highly conserved residue appears in high-consensus red color and as an uppercase letter in the consensus line while a weakly conserved residue is colored blue and as a lowercase letter. A position with no conserved residue is represented by a dot in the consensus line. Symbol ! represents I and V , $ for L and M , % for F and Y , # for N, D, Q, and E. (*Bottom*) An amino acid sequence alignment of MACV GP1, SABV SPH114202 GP1, and SABV GP1 from SP17. The sequence consensus and color scheme are as the top panel. Both alignments were plotted using MultAlin [(13)](https://paperpile.com/c/EQ6D8X/SRNVI).


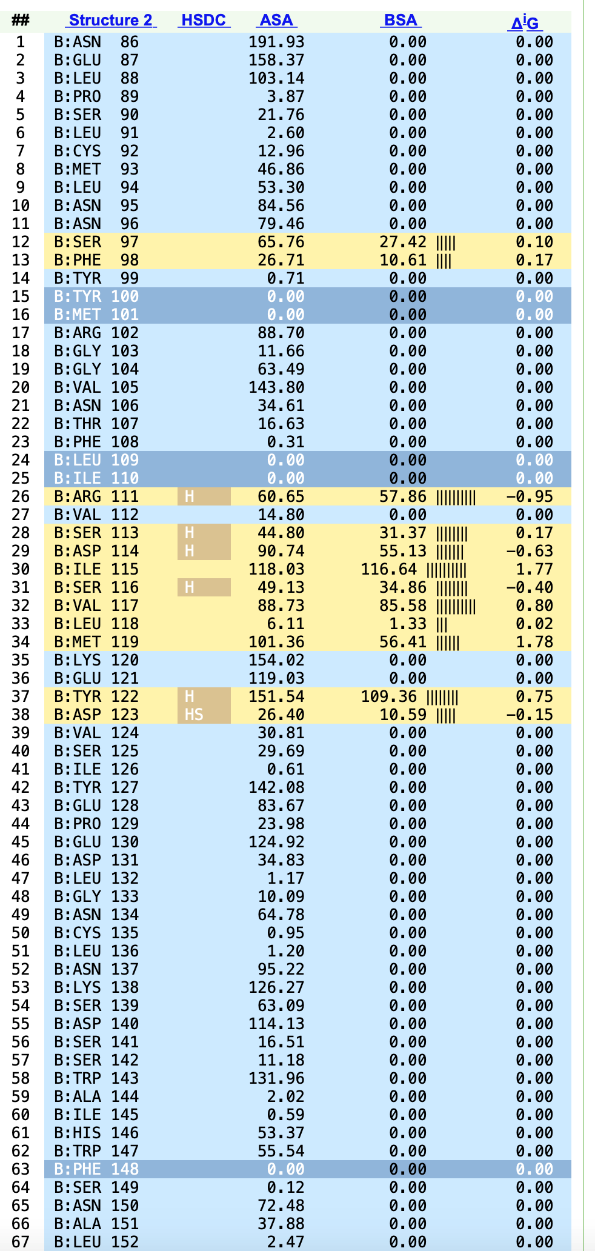

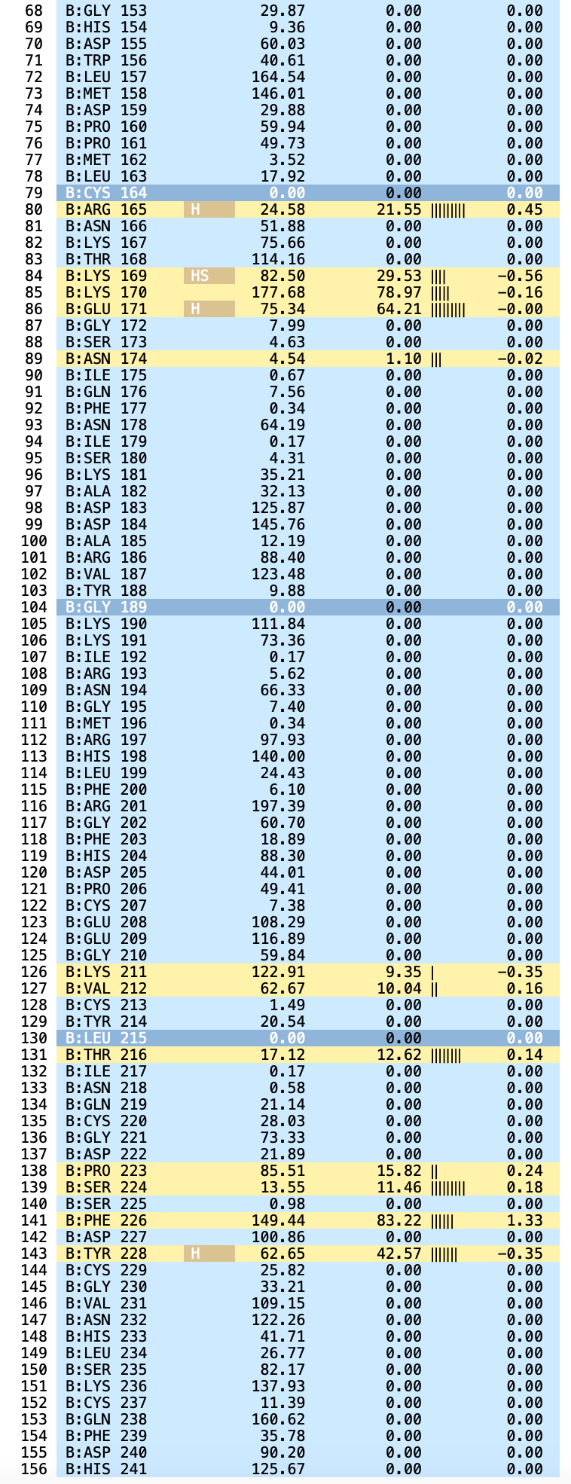


**Fig F. Analysis of MACV GP1 residues that interact with hTfR1.** The structure of MACV GP1 in complex with hTfR1 (PDB no. 3KAS) was submitted to PDBePISA to analyze the interaction interface. The calculated results show accessible/buried surface area (ASA/BSA; Å^2^), solvation energy effect (Δ^i^G; kcal/mol), and intermolecular interactions (HSDC stands for hydrogen bond, salt bridge, disulphide bond and covalent link). Buried area percentage is represented by vertical bars (one bar per 10%). Residues on light blue background are solvent accessible residues while those on a darker blue shade are inaccessible residues. Only MACV GP1 (“Structure 2”) residues 86–241 are shown here.

**Tables**

**Table A. Epidemiological characteristics of eight YFV-negative cases.** ID = identity; Patients were also previously tested negative for the viruses dengue, hepatovirus A, B, C Delta, and E, HIV, CMV, EBV, and HSV, as well as malaria, leptospirosis, toxoplasmosis, and bacterial septic shock.

| **ID** | **Symptom onset** | **Collection date** | **Age** | **Sex** | **Municipality** | **Outcome** | **Metagenomic result** |
| --- | --- | --- | --- | --- | --- | --- | --- |
| SP1 | 2018-03-23 | 2018-04-02 | 21 | Male | São Paulo | Discharged | No clear cause found |
| SP2 | 2018-03-08 | 2018-03-15 | 19 | Female | Cotia | Discharged | No clear cause found |
| SP3 | 2019-01-21 | 2019-01-24 | 31 | Female | Jacupiranga | Discharged | No clear cause found |
| SP4 | 2018-03-22 | 2018-04-02 | 36 | Male | São Paulo | Discharged | Hepatovirus A (HAV) |
| SP5 | 2019-10-31 | 2019-11-09 | 26 | Female | São Paulo | Discharged | No clear cause found |
| SP17 | 2019-12-30 | 2020-01-06 | 52 | Male | Sorocaba | Death 2020-01-11 | Brazilian mammarenavirus |
| SP18 | 2018-12-29 | 2019-01-02 | 24 | Female | São Paulo | Discharged | No clear cause found |
| SP19 | 2019-12-01 | 2019-12-10 | 63 | Male | Assis | Death 2019-12-12 | Brazilian mammarenavirus |

**Table B. Epidemiological characteristics of all reported SABV cases.** YFV = Yellow fever virus; USA = United States of America; NA = natural acquired. Cases from Dec 2019 were most likely infected in Assis (420 km from Cotia) and Sorocaba/Eldorado (65/210 km from Cotia). Distance between Assis and Sorocaba or Eldorado is 356/430 km. All patients had a fatal clinical outcome. No genomic report exists of strain Case 2 (SPH185338).

| **Case** | **Month/Year** | **Sex/Age** | **Location** | **Situation** | **Outcome** | **Reference** |
| --- | --- | --- | --- | --- | --- | --- |
| Case 1 | 01/1990 | Female, 25 | Cotia, São Paulo, Brazil | Admitted as YFV case; agricultural engineer; naturally acquired. | Fatal | Coimbra et al. (SPH114202) [(14)](https://paperpile.com/c/EQ6D8X/BqBdq) |
| Non-NA | 08/1992 | Female, 39 | Instituto Evandro Chagas, Belém, Pará, Brazil | Laboratory technician. | Non-fatal | Vasconcelos et al. |
| Non-NA | 08/1994 | Male, 46 | Arbovirus Research Unit, Yale University, New Haven, Connecticut, USA | Laboratory technician;  bucket centrifuge accident. | Non-fatal | Gandsman et al. |
| Case 2 | 05/1999 | Male, 32 | Espírito Santo do Pinhal, São Paulo, Brazil | Coffee-grain machine operator; naturally acquired. | Fatal | Coimbra et al. (SPH185338) [(15)](https://paperpile.com/c/EQ6D8X/mMQrR) |
| Case 3 SP19 | 12/2019 | Male, 63 | Sorocaba, São Paulo, Brazil | Admitted as YFV case; wall painter who had hiked in the forest; naturally acquired. | Fatal | Nastri et al. |
| Case 4 SP17 | 01/2020 | Male, 53 | Assis, São Paulo, Brazil | Admitted as YFV case; resided in a rural area; naturally acquired. | Fatal | de Mello et al. |

**Table C. Sequencing and alignment statistics for the SABV and HAV cases analyzed in this report, including sequencing coverage statistics.**

| **Strain** | **Total  no. reads** | **Aligned reads (unique)** | **Genome segment** | **Sequence coverage  >1X (%)** | **Sequence coverage  ≥ 5X (%)** | **Mean  coverage depth (x)** |
| --- | --- | --- | --- | --- | --- | --- |
| SP4 | 2,037,725 | 22,289 (1.09%) | whole  genome | 100 | 99.17 | 2,309 |
| SP17 | 14,423,445 | 17,320 (0.12%) | S | 100 | 100 | 407 |
|  |  |  | L | 91.6 | 80.1 | 117 |
| SP19 | 6,424,328 | 132 (0.002%) | S | 74.24 | 57.7 | 7.48 |
|  |  |  | L | 34 | 14.2 | 2.57 |

**Table D. Nucleotide and amino acid identity among SABV strains.**

| **Strain** | **Genome segment** | **Identity (%) to given strain (nt/aa)** |
| --- | --- | --- |
| SP17 | L | SP19: 84.53% / 90.27%   SPH114202: 73.59% / 68.2% |
|  | S | SP19: 73.06% / 76.97%  SPH114202: 89.00% / 95.16% |
|  | Nucleoprotein (NP) | SP19: 98.19% aa  SPH114202: 93.05% aa |
| SP19 | L | SPH114202: 87.75% / 94.39% |
|  | S | SPH114202: 74.44% / 77.74% |
|  | Nucleoprotein (NP) | SPH114202: 93.05% aa |

**Table E. SABV primer sets used for validation of an RT-PCR assay.** Primers were designed based on the 1990 reference sequence of SABV and newly generated genomes from this study. Conserved regions of the nucleoprotein (NP) gene were selected as the amplification targets due to their diagnostic relevance and sequence conservation across SABV strains. Degenerate bases were incorporated where variability was observed (e.g. primer set 3 – All_3R; primer set 5 – All_2R).

| **Primer  sets** | **Name** | **Sequence 5’ - 3’** | **Number**  **of bases** | **Amplicon   Length** | **Tm** |
| --- | --- | --- | --- | --- | --- |
| 1 | Pos_29F | GTCACGCTTAAATCTTTGATTGC | 23 | 350 bp | 55°C |
|  | Pos_381R | ACAGACACCTCAAGACACCA | 20 |  |  |
| 2 | Pos_1642_2F | AGTGTGAAGAGCAATGCATCAG | 22 | 200 bp | 52°C |
|  | Pos_1842R | AATCTGGTCAGATTTTGGTCACC | 23 |  |  |
| 3 | All_3F | TCACGCTTAAATCTTTGATTGCG | 23 | 121 bp | 65°C |
|  | All_3R | GCAGCAATYAAGCTCACTGC | 20 |  |  |
| 4 | Pos_2F | CGCACCGGGGATCCTAGGC | 19 | 380 bp | 55°C |
|  | Pos_381R | ACAGACACCTCAAGACACCA | 20 |  |  |
| 5 | All_2F | GCAGTGAGCTTRATTGCTGC | 20 | 723 bp | 60°C |
|  | All_2R | YACTGCATCAGTTATTGTCC | 20 |  |  |
| 6 | Pos_171F | AACCTGTGGAAGAGTGGCCT | 20 | 200 bp | 52°C |
|  | Pos_381R | ACAGACACCTCAAGACACCA | 20 |  |  |

f = forward primer, r = reverse primer, bp = base pairs, Tm = melting temperature.

**Table F. Reagents and conditions for the SABV RT-PCR protocol.**

| **Component** | **Volume in 25 uL reaction** |
| --- | --- |
| 5x Q5 reaction buffer | 5 μL |
| 10 mM dNTPs | 0.5 μL |
| Q5 DNA polymerase | 0.25 μL |
| Nuclease-free water | Up to 25 μL |
| Primers 10 μM | 1.25 μL forward primer  1.25 μL reverse primer |
| cDNA | 2.5 μL |

**Table G. PCR cycling conditions for the SABV RT-PCR detection protocol.**

| **Temperature** | **Time** | **No. cycles** |
| --- | --- | --- |
| 98 ^0^C | 1 min | 1x |
| 98 ^0^C | 30 s | 35 x |
| Variable | 30 s |  |
| 72 ^0^C | 1 min |  |
| 72 ^0^C | 2 min | 1x |
| 4 ^0^C | Hold | |

**Table H. Primer sequences used in the nested-cPCR reactions.**

| **Primer** | **Sequence (5’-3’)** | **Number   of bases** | **Amplicon Length** | **Tm** |
| --- | --- | --- | --- | --- |
| Pos_29F_outer | GTC ACG CTT AAA TCT TTG ATT GC | 23 | 350 bp | 55 ^o^C* |
| Pos_381R_outer | ACA GAC ACC TCA AGA CAC CA | 20 |  |  |
| Pos_171F_inner | AAC CTG TGG AAG AGT GGC CT | 20 | 200 bp | 52 ^o^C* |
| Pos_381R_inner | ACA GAC ACC TCA AGA CAC CA | 20 |  |  |
| S_outer_1_f | TCA GTG CAG GGA CAG ATC CA | 23 | 494 bp | 69 ^o^C* |
| S_outer_1_r | TCC CTG AGA AGA GGG CTC AG | 20 |  |  |
| S_inner_1_f | TAC AAC CCC TGG AGA CCT CA | 20 | 112 bp | 68 ^o^C* |
| S_inner_1_r | TCA GGA GGT GTG TAC CTG GG | 20 |  |  |
| RNAse P-f | AGA TTT GGA CCT GCG AGC G | 19 |  | 65.5 ^o^C |
| RNAse P-r | GAG CGG CTG TCT CCA CAA GT | 20 |  | 67.4 ^o^C |
| RNAse P-p | HEX-TTC TGA CCT /ZEN/ GAA GGC TCT GCG CG | 22 |  | 71.6 ^o^C |

f = forward primer, r = reverse primer, p = probe, bp = base pairs, Tm = melting temperature.

**Table I. Nested-cPCR reagents and conditions for the SABV detection protocols.** Note that the reaction is done twice, once for the outer PCR reaction, and then for the inner PCR reaction.

| **Component** | **Volume in 25 uL reaction** |
| --- | --- |
| 5x Q5 reaction buffer | 5 μL |
| 10 mM dNTPs | 0.5 μL |
| Q5 DNA polymerase | 0.25 μL |
| Nuclease-free water | Up to 25 μL |
| Primers 10 μM | 1 μL forward primer and 1 μL reverse primer |
| cDNA | 2.5 μL |

**Table J**. **PCR cycling conditions for the SABV nested-cPCR detection protocol.** Note that the reaction is done twice, once for the outer PCR reaction, and then for the inner PCR reaction.

| **Temperature** | **Time** | **No. cycles** |
| --- | --- | --- |
| 98 ^0^C | 1 min | 1x |
| 98 ^0^C | 30 s | 35 x |
| Variable | 30 s |  |
| 72 ^0^C | 1 min |  |
| 72 ^0^C | 2 min | 1x |
| 4 ^0^C | Hold | |

**References**

1. [Claro IM, Ramundo MS, Coletti TM, da Silva CAM, Valenca IN, Candido DS, et al. Rapid viral metagenomics using SMART-9N amplification and nanopore sequencing. Wellcome Open Res. 2021;6:241.](http://paperpile.com/b/EQ6D8X/We342)

2. [Wood DE, Lu J, Langmead B. Improved metagenomic analysis with Kraken 2. Genome Biol. 2019 Nov 28;20(1):257.](http://paperpile.com/b/EQ6D8X/6HeJD)

3. [Breitwieser FP, Salzberg SL. Pavian: interactive analysis of metagenomics data for microbiome studies and pathogen identification. Bioinformatics. 2020 Feb 15;36(4):1303–4.](http://paperpile.com/b/EQ6D8X/xwCln)

4. [Li H. Minimap2: pairwise alignment for nucleotide sequences. Bioinformatics. 2018 Sep 15;34(18):3094–100.](http://paperpile.com/b/EQ6D8X/9v6L4)

5. [Li H, Handsaker B, Wysoker A, Fennell T, Ruan J, Homer N, et al. The Sequence Alignment/Map format and SAMtools. Bioinformatics. 2009 Aug 15;25(16):2078–9.](http://paperpile.com/b/EQ6D8X/4CiLT)

6. [Milne I, Bayer M, Cardle L, Shaw P, Stephen G, Wright F, et al. Tablet--next generation sequence assembly visualization. Bioinformatics. 2010 Feb 1;26(3):401–2.](http://paperpile.com/b/EQ6D8X/5QAbb)

7. [Website [Internet]. Available from:](http://paperpile.com/b/EQ6D8X/1dYg6) <https://nanoporetech.github.io/medaka/>

8. [Altschul SF, Gish W, Miller W, Myers EW, Lipman DJ. Basic local alignment search tool. J Mol Biol. 1990 Oct 5;215(3):403–10.](http://paperpile.com/b/EQ6D8X/O6tY)

9. [Website [Internet]. Available from:](http://paperpile.com/b/EQ6D8X/jI9BW) [NCBI Virus. https://www.ncbi.nlm.nih.gov/labs/virus/vssi/ (accessed June 22, 2023).](about:blank)

10. [Katoh K, Standley DM. MAFFT multiple sequence alignment software version 7: improvements in performance and usability. Mol Biol Evol. 2013 Apr;30(4):772–80.](http://paperpile.com/b/EQ6D8X/wEcA2)

11. [Larsson A. AliView: a fast and lightweight alignment viewer and editor for large datasets. Bioinformatics. 2014 Nov 15;30(22):3276–8.](http://paperpile.com/b/EQ6D8X/mutZR)

12. [Minh BQ, Schmidt HA, Chernomor O, Schrempf D, Woodhams MD, von Haeseler A, et al. IQ-TREE 2: New Models and Efficient Methods for Phylogenetic Inference in the Genomic Era. Mol Biol Evol. 2020 May 1;37(5):1530–4.](http://paperpile.com/b/EQ6D8X/aozfj)

13. [Mitchell C. MultAlin–multiple sequence alignment. Bioinformatics. 1993;9(5):614–614.](http://paperpile.com/b/EQ6D8X/SRNVI)

14. [Lisieux T, Coimbra M, Nassar ES, Burattini MN, de Souza LT, Ferreira I, et al. New arenavirus isolated in Brazil. Lancet. 1994 Feb 12;343(8894):391–2.](http://paperpile.com/b/EQ6D8X/BqBdq)

15. [Coimbra TLM, Santos RN, Ferreira IB, Fialho DM, Mello ES, Ferreira LMH, et al. Arenavirus: A fatal outcame [Internet]. Vol. 6, VIRUS Reviews & Research. 2001. Available from:](http://paperpile.com/b/EQ6D8X/mMQrR) <http://dx.doi.org/10.17525/vrrjournal.v6i1.187>
